# The Knowledge, Attitude, and Practice of Lebanese Mothers Toward Their Children’s Oral Health: A Cross-Sectional Survey

**DOI:** 10.7759/cureus.42903

**Published:** 2023-08-03

**Authors:** Antoine Choufani, Rasseel Barakat

**Affiliations:** 1 Pediatric and Public Health Dentistry, Lebanese University, Beirut, LBN; 2 Pediatric Dentistry and Public Health, Private Practice, Windsor, CAN

**Keywords:** mothers' attitude, children, oral health, knowledge attitude and practices related to health seeking, mothers' knowledge

## Abstract

Objectives: This study aims to assess the knowledge, attitude, and practice of Lebanese mothers toward their children’s oral health, examine the association between the three variables, and identify their predictors.

Methods: A cross-sectional online survey was administered to Lebanese mothers residing in Lebanon with children aged six months to 12 years between April and May 2022. The survey was administered to mothers from various geographical areas who presented to academic medical centers, private clinics, and dispensaries. The survey gathered sociodemographic data as well as assessed the knowledge, attitude, and practice of the participating mothers toward their children's oral health.

Results: A total of 357 responses were included in the final analysis. The mean age of mothers was 32.67 years +/- 6.35. The majority of mothers had one or two children (35.8% and 37.5%, respectively). More than two-thirds of the mothers were unemployed. Only 13% of the mothers followed best practices for their children's dental health, while 48.6.2% possessed above-average knowledge and 88.9% had great attitudes. Mothers with a university education had higher knowledge, better attitudes, and practice scores than those with school-level education (elementary, high school). Employed mothers had a significantly better knowledge score (p=0.036) and practice score (p=0.043) than unemployed mothers. The decrease in the number of children was associated with an increase in the maternal knowledge score. An increase in the mother's age was associated with a higher knowledge score.

Conclusion: The findings of this study suggest the need for targeted oral health education programs for Lebanese mothers to enhance their attitudes and practices toward their children's oral health. It highlights the importance of early oral health interventions and emphasizes the significant role of mothers in promoting good oral health practices for their children. Further research is needed on a larger scale to comprehensively understand these variables and inform the development of appropriate national oral health programs for children in Lebanon.

## Introduction

Early childhood caries (ECC) is a severe public health issue that affects 60% to 90% of children between the ages of two and 11 worldwide. It is defined as "the presence of one or more decayed (non-cavitated or cavitated lesions), missing (due to caries), or filled tooth surfaces in any primary tooth in a child 71 months of age or younger" [1]. Early childhood caries could negatively impact children's quality of life as it can cause pain, infection, difficulties chewing food, and a higher tendency to develop dental caries in primary and permanent dentitions [2]. It also affects the child’s weight, growth, and cognitive development [3].

Parents, particularly mothers, are considered the primary and most important caregivers for their children. They play a crucial role in the prevention and treatment of oral diseases in children. Evaluating parental knowledge, attitude, and practice regarding dental hygiene is crucial, as parents’ outlook will ultimately reflect on their children’s oral health. Studies have shown that the knowledge of mothers regarding their children’s oral cavities depends on the parent's education level, interest, and access to necessary information [4]. The existing literature also revealed that mothers' engagement in good dental practices positively impacts children’s oral health [5].

Although dental caries steadily decreased in countries that implemented community prevention programs, disadvantaged communities in both developing and developed countries continue to be affected. According to the World Health Organization (WHO), the dental caries index (decayed, missing, and filled tooth (DMFT)) among Lebanese children aged 12 years has worsened throughout the years [6]. Data from the WHO showed that in 2018, 88% of Lebanese children aged six to eight had a history of dental caries, and 86% still had their primary teeth untreated [6]. Moreover, studies also found that mothers have a substantial impact on their children’s current and future emotional health, personality, character, well-being, social and cognitive development, and academic performance [7]. In Lebanon, however, the knowledge, attitude, and practice of mothers regarding their children's dental health had not previously been researched. A mother’s knowledge and awareness about the risk factors associated with the development of ECC play a vital role in the prevention of the disease [8]. Furthermore, awareness of their children’s oral health and how to maintain good oral hygiene reduces the prevalence of ECC [8]. Encouraging mothers to implement good oral health behaviors and adopt a healthier lifestyle generally results in positive long-term effects for both the mother's and child’s health [9]. This study, therefore, aims to assess Lebanese mothers’ knowledge, attitude, and practice toward their children’s oral health while examining the association between the three variables and identifying their predictors.

## Materials and methods

Study design and setting

A cross-sectional online survey was administered to Lebanese mothers residing in Lebanon with children aged six months to 12 years between April and May 2022. The online survey aimed to explore Lebanese mothers’ perceptions of their children’s oral health. To achieve diversity, potential participants were compiled from various geographical areas, including academic medical centers, private clinics, and dispensaries from both private and public healthcare facilities. The survey was then administered to all eligible mothers. Google Forms (Alphabet Inc., Mountain View, CA, USA), a user-friendly online survey tool, was used to conduct the questionnaire. Individual invitations were sent to potential participants via social media platforms (WhatsApp, Facebook, and Instagram (Meta Platforms, Inc., Melo Park, CA, USA)). Personalized direct messages were used to encourage participation, containing the survey link and relevant information about the study. Prior to proceeding with the questionnaire, participants were required to provide electronic informed consent, ensuring ethical compliance and protecting participants' rights and confidentiality. By using these social media platforms, the researchers aimed to reach a broader and more diverse audience from various geographical areas. The survey questionnaire consisted of 22 questions and aimed to gather data on oral health practices, knowledge, and behaviors of the mothers in relation to their children's oral health. Completing the questionnaire took approximately 15 minutes. To uphold privacy and anonymity, the researchers implemented measures to secure the data and separate personal identifying information from survey responses. Furthermore, the study received ethical approval from the Institutional Review Board at the Lebanese University, Faculty of Dental Medicine, ensuring adherence to ethical guidelines.

Sample size

A sample size calculation was carried out considering the primary outcome and based on a previous study that reported that 43.7% of participants have high oral health knowledge [10]. A sample of 347 mothers was estimated with a 90% CI and a 5% margin of error. Additional participants were recruited to account for potential exclusions during the analysis phase [10]. Population size was considered very large (more than 1 million), and the following formula was used: statistical power is the probability of correctly identifying a positive hypothesis (true positive), hence rightfully rejecting the null hypothesis. This is completely different from the confidence level. A power of 90% is considered very high and means that the hypothesis tests in the analysis are powerful enough, and leave less room for type II error.

LaTeX Format

\\ N = \frac{p_0(1-p_0) \Bigg\{ z_{1-\alpha/2} + z_{1-\beta} \sqrt{\frac{p_1(1-p_1)}{p_0(1-p_0)}} \Bigg\}^2 }{(p_1-p_0)^2}

Measurements

The questionnaire was constructed based on a review of the published peer-reviewed literature and other surveys examining oral health knowledge. A preliminary version of the survey was constructed upon this review, which was reviewed by a group of specialists in pediatric dentistry, public health, dental public health, and methodology. The expert team modified the questionnaire as necessary, and a final consensus was reached on the final version, which was then pilot-tested on a number of Lebanese mothers. The questionnaire was developed in Arabic, as all Lebanese mothers are fluent in Arabic.

The questionnaire included basic demographic information about the parents (age, number of children, education, and occupation). The questionnaire also included multiple-choice questions to assess the knowledge, attitude, and practice of mothers towards their children's oral health. To assess a mother’s knowledge about children’s oral health, seven questions were asked. For each correct response, participants scored one point out of seven, and for each incorrect answer, no point was gained. The maximum knowledge score participants can get is seven, and the lowest score is zero.

To assess mothers practices toward their children’s oral health, six questions were asked. Questions were scored similarly to the knowledge score. To assess mothers’ attitudes toward their children’s oral health, participants were asked whether they agreed or disagreed with five statements. Attitude does not have a definitive right or wrong answer; it can either be positive or negative. In the questionnaire, the response options for attitude are as follows: "agree" is assigned a score of 1, "don't agree" is assigned a score of 2, and "not sure" is assigned a score of 3. By summing the scores of the attitude questions, a total score of 5 indicates that the participant selected "agree" for all questions, indicating a positive attitude. On the other hand, a total score of 15 indicates that the participant chose "don't agree" for all questions, reflecting a negative attitude. Consequently, a lower attitude score, such as 5, indicates a more positive attitude, while a higher attitude score, like 15, indicates a more negative attitude. The scores were categorized according to Bloom’s cut-offs (0% to 59%, 60% to 79%, 80% to 100%); the maximum score refers to a better level, except for attitude, where a low score (i.e., 5) refers to a better attitude and a high score (i.e., 15) refers to a bad attitude towards the child’s oral health.

Statistical analysis

The SPSS Statistics version 25.0 (IBM Corp., Armonk, NY, USA) and R Statistical Framework 4.1.2/RStudio 1.4.17 (Rstudio, Boston, MA, USA) were used for data cleaning, management, and analyses. Continuous and categorical variables were described as mean and standard deviation and frequency and percentages, respectively. Graphical quantile plots were used to evaluate the normality of ages, knowledge score, attitude score, and practice score. Depending on each variable distribution and respective assumptions, bivariate analysis was performed using the Chi-square test, independent samples T-test, one-way, and Welch's. The ANOVA, Mann-Whitney U test, and Kruskal Wallis test were also used along with post-hoc tests following the ANOVA and Kruskal Wallis tests to evaluate the level of difference among educational levels and professional status (Tukey honestly significant difference (HSD) following the ANOVA test and pairwise Mann-Whitney U with Bonferroni correction following the Kruskal Wallis test). Internal consistency of knowledge, attitude, and practice scales was evaluated with a split-half method using the "psych" and "ltm" libraries. Finally, linear regression modeling was used to map predictors of said scales and to adjust for included confounders. All reported p-values refer to two-sided tests, and statistical significance was set below an alpha of 5%. 

## Results

A total of 360 mothers were recruited for this study, out of which three were excluded for not having children aged six months to 12 years. Table 1 presents the baseline characteristics of the parents. The mean age of mothers was 32.67 years +/- 6.35. The majority of mothers had one or two children (35.8% and 37.5%, respectively). Half of the mothers were university graduates (50.6%), whereas 40.6% of the fathers were university graduates. More than two-thirds of mothers were unemployed (64.2%), whereas nearly half of the fathers were employed (49.2%).

**Table 1:** Demographic characteristics of parents

Characteristics		Mean (SD)	
Age of mother		32.67 years (6.354)	
Age of father		38.72 years (7.158)	
		N	Percentage
Mother’s educational level	Elementary school	75	20.8
	High school	98	27.2
	University	182	50.6
	No formal education	5	1.4
Father’s educational level	Elementary school	106	29.4
	High school	92	25.6
	University	146	40.6
	No formal education	16	4.4
Mother’s professional status	Businesswoman/entrepreneur	2	0.6
	Student	8	2.2
	Unemployed	231	64.2
	Self-employed	40	11.1
	Employee	79	21.9
Father’s professional status	Businessman/entrepreneur	15	4.2
	Student	1	0.3
	Unemployed	38	10.6
	Self-employed	177	49.2
	Employee	129	35.8
Number of children	No children	3	0.8
	1 child	129	35.8
	2 children	135	37.5
	3 children	67	18.6
	4 children	24	6.7
	5 children	2	0.6
Total		360	100

Table 2 presents the mothers’ knowledge of their children’s oral health. Around 40.3% of mothers reported their child to have 20 milk teeth; the majority responded that toothpaste contains fluoride (71.4%) and that fluoride is essential to prevent tooth decay (67.2%). The majority (93.3%) of mothers reported tooth decay to be the most common dental problem in children, 95.8% reported sugar to be the main cause of tooth decay, and 85.0% reported that milk teeth should be preserved until they fall out naturally. The majority of mothers (66.9%) reported that a combination of the following actions is important to prevent tooth decay: regular visits to the dentist, constant brushing of teeth, and limiting sugar intake.

**Table 2:** Knowledge questionnaire

Questions	Multiple-choice answers	N	Percentage
How many milk teeth are there in a child's mouth when complete?	10	12	3.3
	12	34	9.4
	20	145	40.3
	28	37	10.3
	Don’t know	132	36.7
Does toothpaste contain fluoride?	No	26	7.2
	Yes	257	71.4
	Don't know	77	21.4
What is the role of fluoride?	Refreshes	13	3.6
	Prevents teetth decay	242	67.2
	Prevents gum complications	22	6.1
	Don’t know	83	23.1
What are the most common dental problems in children?	Tooth decay	336	93.3
	Tooth discoloration	15	4.2
	Gum bleeding	5	1.4
	Don’t know	4	1.1
Which of the following nutrients leads to tooth decay?	All of the above	12	3.3
	Sugar	345	95.8
	Vegetables	0	0
	Cheese	0	0
	Meat	0	0
	Don’t know	3	0.8
Which of the following do you think contributes to reducing tooth decay? (You can choose more than one option)	Visit dentist regularly	16	4.4
	Brush teeth	60	16.7
	Limit sugar intake	71	19.7
	All of the above	241	66.9
	Don’t know	6	1.7
Should milk teeth be preserved until they fall naturally?	Certainly	306	85
	Don’t know	13	3.6
	Not necessarily	41	11.4
Total		360	100

Table 3 presents the attitudes of mothers towards their children’s oral health. The majority of mothers acknowledged the importance of regular dental visits (78.1%), the need to help and directly supervise the process of the child cleaning his/her teeth (85.3%), and the need to clean one’s teeth after each meal (64.2%). In addition, the majority of mothers believed that oral and dental problems affect general health (66.7%) and that healthy milky teeth are important for children to properly chew food (77.8%).

**Table 3:** Attitude questionnaire

Statements	Agree, n (percentage)	Not sure, n (percentage)	Disagree, n (percentage)
It is important to take your child to the dentist regularly	281	35	44
	(78.1)	(9.7)	(12.2)
Parents should help and directly supervise the child brushing his/her teeth	307	13	40
	(85.3)	(3.6)	(11.1)
It is necessary to clean the child's teeth after each meal	231	75	54
	(64.2)	(20.8)	(15)
Oral and dental problems affect general health	240	77	43
	(66.7)	(21.4)	(11.9)
Healthy milk teeth are important for children to chew food well	280	47	33
	(77.8)	(13.1)	(9.2)

Table 4 presents practices adopted by mothers for their children’s oral health. Around 42.5% reported not visiting the dentist yet, 58.3% reported visiting the dentist only when problems appeared, and 43.6% reported brushing their children’s teeth when all their milk teeth had appeared. About 43.6% reported brushing their child’s teeth regularly, 45% reported changing their child’s toothbrush every two to three months, and half of them reported that their child does not consume sugar regularly (51.4%). 

**Table 4:** Questionnaire to determine practices adopted by mothers to maintain their children’s oral health

Questions	Multiple-choice answers	N	Percentage
When was your child's first visit to the dentist?	1 year after the child’s birth	99	27.5
	6 months after the child’s birth	12	3.3
	After the appearance of the first milk tooth	96	26.7
	Haven’t visited yet	153	42.5
When do you take your child to visit the dentist?	When problems appear	210	58.3
	Every 6 months	60	16.7
	Yearly	23	6.4
	Not regularly	67	18.6
When did you start brushing your child's teeth?	4 to 6 months after the appearance of milk teeth	48	13.3
	When the first milk tooth appears	81	22.5
	When all milk teeth appear	157	43.6
	Don’t remember	74	20.6
How often do you brush your child's teeth?	After each meal	30	8.3
	Not regularly	78	21.7
	Once daily	95	26.4
	Twice daily	157	43.6
When do you change your child's toothbrush?	As soon as the brush bristles are bent	94	26.1
	Every 2 to 3 months	162	45
	Not regularly	104	28.9
What time does your child eat sugar?	Between meals	162	45
	Before bedtime	1	0.3
	Not regularly	185	51.4
	With meals	12	3.3
Total		360	100

Table 5 presents the characteristics of knowledge, attitude, and practice indices. Half of the sample had a high knowledge level (48.6%), with a mean score of 5.2 ± 1.5; yet a third of the mothers had a low level of knowledge (30.3%). As for the attitude, the majority of the mothers showed a positive attitude (88.9%) with a mean score of 6.9±2.8. Surprisingly, 84.2% of mothers reported poor child oral health practices, with a mean score of 1.4 ± 1.1.

**Table 5:** Characteristics of knowledge, attitude, and practice scales indices

Variables	Level	N	%	Score mean ± SD	Score range
Knowledge	Low	109	30.3	5.2 ± 1.5	0-7
	Intermediate	76	21.1		
	High	175	48.6		
Attitude	Good	320	88.9	6.9 ± 2.8	5-15
	Moderate	7	1.9		
	Poor	33	9.2		
Practice	Poor practice	303	84.2	1.4 ± 1.1	0-5
	Average practice	44	12.2		
	Good practice	13	3.6		
	Total	360	100		

Linear regression analysis was performed on knowledge, practice, and attitude scores; the best models were selected based on model indicators (goodness of fit, R2, and Durbin-Watson statistic). Relevant factors were included in models based on theoretical backgrounds rather than on arbitrary automated methods (Table 6).

**Table 6:** Linear regression model, dependent variable:practice score P<0.001, R2=9.2%, D-W=2.062

Model 1	B	Standard error	Standardized beta	t	p	95.0% Confidence interval for B	
Constant	1.197	0.438		2.731	0.007	0.335	2.059
Knowledge score	0.197	0.040	0.258	4.918	0.000	0.118	0.275
Children count	-0.109	0.066	-0.092	-1.648	0.100	-0.239	0.021
Mother’s age	-0.015	0.016	-0.084	-0.922	0.357	-0.047	0.017
Lowest child age	0.015	0.022	0.042	0.660	0.510	-0.029	0.059

Neither the mother's nor the lowest child's age affected the practice of mothers. Moreover, there was no significant relationship between children’s count and the practices of mothers toward their children’s oral health. The practice score appeared to be influenced by the knowledge score. Each 10-point increase in knowledge score was associated with an increase of 2 points in practice. Therefore, better maternal knowledge seems to lead to better oral health practices in children.

As expected, a better attitude (a decreased attitude score of 5 indicating the best attitude and 15 the poorest) was associated with an increased knowledge score. Moreover, a decrease in the number of children was associated with an increase in the maternal knowledge score, while an increase in the mother’s age was predictive of the increased knowledge score (Table 7). As for attitude, the main finding in regression analysis was that an increased knowledge score was positively linked to a 'decreased' attitude score and hence a better attitude (p<0.0001, B=-0.638, R2=12.7%, D-W=1.952).

**Table 7:** Linear regression model, dependent variable:knowledge score P<0.001, R2=15.4%, D-W=1.894

Model 2	B	Standard error	Standardized beta	t	p	95.0% Confidence interval for B	
Constant	7.004	0.484		14.472	<0.0001	6.052	7.955
Children count	-0.225	0.083	-0.144	-2.709	0.007	-0.388	-0.062
Mother’s age	0.051	0.021	0.217	2.487	0.013	0.011	0.092
Lowest child age	-0.044	0.028	-0.096	-1.563	0.119	-0.1	0.011
Attitude	-0.17	0.026	-0.325	-6.514	<0.0001	-0.221	-0.118

## Discussion

This is a first-of-its-kind study to assess Lebanese mothers' knowledge, attitude, and practice regarding their children's dental health. The findings of this study showed that only a small percentage of mothers followed best practices for their children's dental health, 48.6.2% of mothers possessed above-average knowledge, and the majority had great attitudes. Mothers with a university education had higher knowledge, attitude, and practice scores than those with a school-level education. Employment status, a decrease in the number of children, and a mother’s age were significantly associated with a higher knowledge and practice score.

Studies have shown that mothers' dental knowledge significantly influences their children's oral health and oral health-related behaviors [1,2,11]. Our findings reveal that nearly half of the mothers had a high knowledge level (48.6%) with the majority reporting a positive attitude (88.9%). Surprisingly, only 13% of mothers reported good child oral health practices. These results suggest that a positive attitude on its own is not sufficient to implement good dental practices.

The findings of our study concur with the literature that reports mothers with university education levels have better oral health knowledge as compared to those with school-level education [1]. This can be justified by the inference that mothers with a lower education level may be unaware of the effects of potential risk factors associated with the development of oral diseases. Therefore, health education and promotion are crucial for mothers with limited educational backgrounds [12]. Our findings are also in line with the literature that showed that high levels of knowledge among mothers led to better oral health practices among their children [13]. Our findings also showed that a positive attitude is associated with an increased knowledge score. This is also in line with the literature [14].

This study indicated that a decrease in the number of children was associated with an increase in the maternal knowledge score, while an increase in the mother’s age was predictive of an increased knowledge score. These results concur with those reported by Alshammari et al., where the number of children was negatively correlated with maternal knowledge [15]. Contrarily, a study by Abduljalil et al. reported that there was no significant correlation between knowledge, the mother’s age, and the number of children in the household [2]. 

The findings of our study revealed noteworthy insights into the relationship between maternal employment status and maternal knowledge and practices regarding their children's oral health. According to the results, employed mothers exhibited significantly higher knowledge scores (p=0.036) and practice scores (p=0.043) than unemployed mothers. This suggests that maternal employment may be associated with a better understanding of oral health-related information and more favorable oral health practices in their children's daily routines. Our results align with previous research, reinforcing the idea that maternal employment can have a positive impact on children's oral health outcomes. In a study conducted by Vieira-Andrade et al., similar patterns were observed. They reported that children living with employed caregivers and those with higher wages were less likely to experience caries, indicating a potential protective effect of maternal employment on children's oral health [1,16]. The observed positive association between maternal employment and maternal knowledge and practices regarding their children's oral health could potentially be attributed to multiple factors. One plausible explanation is the increased household income that comes with employment, allowing families to afford better care for their children's oral health needs. Employed mothers may have greater financial resources to invest in preventive measures, such as regular dental check-ups, fluoride treatments, and healthier dietary choices for their children. This financial stability might lead to a higher likelihood of seeking professional dental care, which, in turn, can positively impact the children's oral health outcomes. Moreover, the association could be influenced indirectly by the educational level of the mother. It is possible that employed mothers, on average, possess higher levels of education compared to unemployed mothers. Higher educational attainment is often linked to better health literacy and an increased understanding of preventive health practices. Therefore, employed mothers with higher educational levels may be more inclined to seek and absorb oral health-related information, leading to improved knowledge and better oral health practices for their children. Further investigation through more in-depth research is needed. The relationship between maternal employment, household income, educational levels, and their combined impact on children's oral health is likely to be complex and multifaceted.

Our findings showed that 40.3% of mothers correctly identified the number of teeth. These findings, however, do not indicate the source of the information used to answer the question. More than two-thirds of mothers (77.8%) believed that primary teeth are necessary for children to chew food effectively and that good oral health is linked to overall health. Our findings contradict those reported by Mahmoud et al., who found that only 22.2% of mothers knew the exact number of milk teeth yet had an exceptional attitude (99.9%) toward the importance of teeth for chewing [1].

Around 71% of mothers admitted that the toothpaste they use for their children contains fluoride, and more than two-thirds of them acknowledged the importance of fluoridated toothpaste in preventing dental caries. These results are satisfactory, as the use of fluoridated toothpaste is considered a safe, easy, and cost-effective method that greatly prevents dental caries. These results are in accordance with those reported by Kashyap et al., where almost two-thirds of mothers used fluoridated toothpaste and acknowledged its importance in preserving their children’s teeth [17]. Conversely, Shilpa et al. reported that the majority of mothers (96.6%) were uninformed of the importance of fluorides in caries prevention [7]. The acknowledgment of the importance of fluoridated toothpaste by more than two-thirds of the surveyed mothers further reinforces the significance of this preventive strategy. Fluoridated toothpaste not only helps strengthen the tooth enamel, making it more resistant to acid attacks from bacteria, but it can also promote the remineralization of early caries lesions, halting the progression of decay. These benefits contribute to the reduction of dental caries, a prevalent oral health issue among children. The high percentage of mothers using fluoridated toothpaste in our study is promising for oral health outcomes. Regular and consistent use of fluoride-containing toothpaste, coupled with proper oral hygiene practices, can significantly lower the risk of dental caries and promote better oral health in children. 

One limitation of our study is that we did not inquire about the specific fluoride concentration in the toothpaste used by the participants. This could have provided valuable insights into the effectiveness of the preventive measures employed. While the findings are encouraging, it is essential to recognize that the concentration of fluoride in toothpaste also plays a vital role in its efficacy and safety. The optimal fluoride concentration in toothpaste, recommended by dental associations and health authorities, ensures effectiveness without posing risks of fluorosis or other adverse effects. Understanding the appropriate fluoride concentration is crucial for mothers to make informed decisions when selecting toothpaste for their children.

Moreover, the results of our study showed that over half of the mothers (42.5%) had not attempted to take their child to a dentist; 27.5% took their child to a dentist one year after childbirth; 3.3% after six months; and 26.7% after the first tooth appeared. These results are in accordance with the existing literature that shows that only a small percentage of mothers established a dental home and took their child to their first dental examination at the age of 12 months [17-19]. These findings highlight the importance of education in preventing ECC. Dental homes must be established no later than 12 months of age, as they will provide dietary counseling, information about the child's oral hygiene practices, acute care and preventive service, an individualized preventive dental health program based on a caries-risk assessment, a periodontal disease risk assessment, and anticipatory guidance [20].

The majority of mothers (78%) believed that taking their children frequently to dental checkups was extremely important. Surprisingly, more than half of the parents only bring their children to dental clinics when they are experiencing pain or discomfort, and less than one-third are considered to do so on a regular basis. This is alarming; however, these findings concur with existing nationwide studies [1,5,21,22]. Regular dental checkups are crucial to enhance the likelihood of recognizing and treating oral diseases in their initial phases and to prevent possible serious or irreversible damage to the teeth [23]. The low socioeconomic status of caregivers and decreased family income were previously highlighted as major reasons for such behavior [21]. A critical review conducted in 2019 demonstrated that socioeconomic status is negatively associated with oral health and dental illness, i.e., the higher one's socioeconomic status, the better one's perspective of oral health, and the fewer clinically diagnosed dental problems one experiences [24]. In our study, however, parental income was not captured; therefore, it is worth examining socioeconomic status as a predictive risk factor in future research.

Furthermore, with the Beirut Blast explosion that took place on August 7, 2020, and the unprecedented pandemic, Lebanon has been undergoing economic turmoil that has further deteriorated Lebanese citizens’ socioeconomic status. This could explain the reasons behind limiting access to oral health services when needed in Lebanon. 

Our findings also showed that 95.08% of mothers were aware of the increased dental caries risk associated with the consumption of sugary snacks. These results are in line with existing studies [17,25,26]. Although mothers were aware of the harmful dental impact associated with consuming sugary snacks, most mothers remained unaware of the significance of the appropriate time for children to consume sugar. Around 45% offer sugary snacks between meals, while only 3.3% limit the intake of sugary foods during the meal. A study by Mahmoud et al. revealed similar findings, whereas another study by Blinkhorn et al. found that 78% of parents limited their children’s sugary intake to mealtimes alone [1,27].

More than two-thirds of mothers positively responded when asked about tooth decay prevention strategies. The majority believed that a multi-method strategy should be used to prevent tooth decay. Sugar restriction is crucial to caries prevention. However, in recent decades, eating patterns have shifted worldwide. Processed food (high in energy, sugar, and salt but low in nutrients), fast food, and beverages have become more popular. The younger generation’s consumption of processed carbohydrates and fats has therefore increased [28]. The majority of mothers also acknowledged the importance of teeth brushing, frequent dental checkups, and fluoride toothpaste in the prevention of dental caries.

Existing literature showed that children whose mothers began brushing their teeth before the age of two had lower DMFT scores as compared to children whose mothers began brushing their teeth after the age of two [29]. Our findings showed that 22.5% of mothers began brushing their children’s teeth immediately after the eruption of the first primary tooth. These findings outperform those of Elidrissi et al. in Sudan, where only 6.8% of mothers indicated brushing their child’s teeth immediately after the first tooth appeared. Another study conducted by Rigo et al. reported satisfying results: 72.2% of parents began cleaning their children's teeth when the first tooth erupted [29,30].

Furthermore, our findings showed a favorable attitude among mothers who supervise their children while brushing their teeth; 85.3% of mothers believed they should do so. These results are consistent with the existing literature [17]. In addition, 43.6% of mothers reported brushing their children’s teeth twice per day, while 21.7% reported not brushing their children’s teeth regularly. These results are comparable to those reported by Jain et al., where 41% of mothers reported brushing their children’s teeth twice a day. These results are disappointing, as brushing is the most cost-effective way to prevent dental caries. According to the American Academy of Pediatric Dentistry (AAPD), toothbrushes must be replaced every three months and should be changed sooner once the bristles start fraying [15]. In our study, 45% of mothers replaced their child’s toothbrush every two to three months, 26.1% replaced it after the bristles frayed, and 28.9% did so on an irregular basis. Comparable results were obtained by a study done on Indian mothers in 2014 [15].

Limitations

This study should be considered in light of its limitations. First, the non-probability and unstratified sampling methods used in the study could be considered a limitation as it may introduce bias and affect the representativeness of the sample. This means that the findings of the study may not be generalizable to the entire population. Regression analyses were conducted to control for confounders. This is a strength, as regression analyses can help identify and account for potential confounding variables, therefore increasing the internal validity of the study and providing a more reliable and accurate estimate of the relationships between the variables of interest. Since the study relied on self-reported data from participants, there was a risk of self-reporting bias. Respondents may have provided answers that they believe are socially desirable. This can lead to inaccuracies and may impact the validity of the results. Moreover, the sample size used in the study is not representative of the entire population of Lebanese mothers. Future studies with larger sample sizes are needed, as it would increase the statistical power of the study and improve the reliability of the findings. Although researchers attempted to gather data from various geographical regions, it is unclear if certain governorates were underrepresented or overrepresented. Moreover, questions to determine participants’ socioeconomic status (income) were not included in the survey. However, by taking into account the aforementioned limitations, the findings of this study set the ground for larger-scale studies aimed at improving children’s oral health.

## Conclusions

This study evaluated the knowledge, attitude, and practice of a specific group of Lebanese mothers concerning their children's oral health. The study also sought to identify the factors contributing to improved oral health in children. The findings of this study imply a necessity for oral health education programs targeted at Lebanese mothers to enhance their attitudes and practices towards their children's oral health. The study also emphasizes the significance of early oral health interventions and the crucial role of mothers in promoting good oral health practices for their children. Lastly, this study contributes to the existing literature by adding to the growing body of research on maternal knowledge, attitudes, and practices regarding children's oral health. It aligns with previous findings that indicate a gap between maternal knowledge and the actual oral health practices of their children. The study indirectly underscores the importance of tailored oral health education programs for mothers, a recommendation that has been made in other studies as well. Further research is required to develop a comprehensive understanding of the three variables studied (knowledge, attitude, and practice) in a larger, nationwide representative study. This will enable the development of appropriate national oral health programs aimed at improving the oral health of children in Lebanon.
